# Supplementary material for: Ethylene: A Master Regulator of Salinity Stress Tolerance in Plants
Source: Biomolecules. 2020 Jun 25;10(6):959. doi: 10.3390/biom10060959 (PMC7355584; doi:10.3390/biom10060959)
Supplement: Supplementary file 1 [file biomolecules-10-00959-s001.zip › biomolecules-837361-supplementary.docx]

**Table S1.** An overview of the recent studies on the role of ethylene in salt-treated plants.

| **Plant** | **Tissue utilized** | **Experimental design/approach** | **Major outcome** | **Response** | **References** |
| --- | --- | --- | --- | --- | --- |
| Arabidopsis | Seeds, shoots and roots | *ein2-5, ein3-1, ctr1-1* mutants | Increase salinity tolerance | + | [47] |
|  | Wild type Col-0 | Wild type Col-0 | Supressed ETR1 expression |  | [56] |
|  | Seedlings | *etr* loss-of-function mutant | Enhanced salt tolerance |  | [55, 57, 58] |
|  | Seedlings | *etr*-1 gain-of-function mutants | Increased sensitivity to salinity stress |  | [55, 57, 58] |
|  | Seedlings | Loss of function of *ein2* | Enhanced sensitivity to salinity stres | - | [55/65] |
|  | Seedlings | Overexpression of *ein2* | Reduced sensitivity to salt stress | + | [55/65] |
|  | Germinating seeds and seedlings | *ecip1* loss- of-function mutants | Sensitivity to salinity stress | - | [65] |
|  | Seedlings | Overexpression of *MnEIL3* | Upregulation of ethylene biosynthetic genes leading to salt stress tolerance | + | [71] |
|  | Leaves | *AP2/ERF* gene (*IbRAP2-12)* | Upregulation of multiple genes involved in ROS-detoxification including *SAPX*, *GPX7*, *CAT5* | + | [80] |
|  | Germinating seeds  Seed germination and seedlings | etr1 and etr2 loss-of-function mutants | Altered seed germination | +/- | [59] |
|  |  | Ectopic expression of *GhPLATZ1* | Regulation of seed germination and seedling establishment | + | [100] |
|  |  | Ectopic expression of *AtERF38* (GhERF38 from *G. hirsutum*) | ABA sensitivity and reduced seed germination under salinity and drought stress | - | [101] |
|  | Shoots | *ctr1-1* mutants, *ein2-5* or *ein3* | Slight reduction in leaf area and root elongation | + | [63] |
|  | Shoots and roots | *Enterobacter* *sp*. SA187 treatment | Salinity tolerance | + | [123] |
|  | Cells and seedlings | Expression of two cysteine protease inhibitors, *AtCYSa* and *AtCYSb* | Improved salinity tolerance | + | [145] |
|  | Roots | *ein3-*1 deficient mutant | Enhanced production of JA | + | [173] |
| Rice | Seedlings | *maohuzi6 (mhz6) mutant*  *MHZ6/OsEIL1* | Salinity hypersensitivity | ­­­­- | [51, 53] |
|  | Superior and inferior spikelets | LYP9 and NPBA cultivars | Reduced growth, grain filling, and development of spikelets | - | [53] |
|  | Rice spikelet | LYP9 and NPBA cultivars | Improved physiological, agronomical, and biochemical characteristics | + | [54] |
|  | Leaves | Overexpression of *OsARD1* in rice variety Zhonghua11 | Increased water holding capacity, relative water content anc increased ethylene concentration | + | [87] |
|  | Seedlings | Overexpression of *OsEIL2* gene | Retarted growth, shorter roots and shoots, increased ethylene sensitivity and accelerated leaf senescence | - | [70] |
|  | Roots and shoots | Dongjinchalbyeo (DJC, salt‐tolerant) and Dongjinbyeo (DJ, salt‐sensitive) cultivers | Salinity tolerance | + | [171] |
| Maize | Leaves and root | Salt-sensitive (BR5011) and salt-tolerant  (BR5033) genotypes | Increase salinity tolerance | + | [12] |
| Grapevine | Leaves | ‘Crimson seedless’  grapevines | Enhanced salinity stress tolerance | + | [13] |
| *Solanum chilense* | Leaves | *Solanum chilense* (accession LA4107) and of cultivated glycophyte *Solanum lycopersicum* (cv. Ailsa Craig) | Effective in adaptation to salinity stress | + | [48] |
| Tomato | Leaves | Tomato plants (*Solanum lycopersicum* L.) cv. Santa Clara | Improved photosynthesis, metabolic homeostasis, and growth rate and lowered amount of ABA hormone and ACC | + | [115] |
|  | Seedlings | *diageotropica* (*dgt*), tomato mutant with reduced IAA sensitivity | Withstand cadmium stress | + | [156] |
| Pinus | Plants | Pinus pinaster Ait seeds from two different population gredos and Bajo Tiétar | Salinity tolerance | + | [170] |
| Cotton |  | Two upland cotton (*G. hirsutum*) genotypes: salt-tolerant Earlistaple 7 and salt-sensitive Nan Dan Ba Di Da Hua | Upregulation of different sets of genes of ethylene signaling involved in the regulation of salinity stress | + | [55] |
| Tobacco |  | *NTHK1* transgenic lines | Negative regulation of ethylene in salinity stress tolerance |  | [57] |
| Tobacco | Germinating seeds | Overexpression of *MhSHN1* gene | Enhances salinity and osmotic stress tolerance | + | [96] |
|  | Roots and leaves | Expression of *LchERF* from *Lycium chinense* in tobacco | Salinity tolerance | + | [105] |
|  | Seedlings | Overexpression of *NTHK1* gene | Alters plant responses to salinity stress |  | [60] |
| *Cynanchum* *auriculatum* | Leaves | Molecular changes of *C. auriculatum* to saline environments | Upregulation of ERF1/2 and downregulation of CTR1 and EBF1/2 |  | [14] |
| Wheat | Leaves | Overexpression of ethylene-responsive transcription factor (TdSHN1) | Development of a cuticle and lower stomatal density | + | [81] |
|  | Germinating seeds | Treatment with *Pseudomonas* *fluorescens* strains | Improved salinity tolerance | + | [102] |
| Poplar | Seedlings | ERF gene, *ERF38* (Potri.006G138900.1), from the 84K poplar (*Populus alba* × *Populus glandulosa*) | Decreased membrane lipid peroxidation, ROS, proline and soluble proteins accumulation and higher POD and SOD activities | + | [83] |
| *Cucumis* *sativus* L. | Germinating seeds | Effect of Glu and ethylene on seed germination and radicle growth of cucumber Seeds | Suppression of seed germination | - | [95] |
|  |  | Exogenous application of BR | Increased levels of ethylene and alternative oxidase pathway (AOX) | + | [174] |
|  |  | CaC_2_ enhancing the activity of SOD and CAT and reducing the H_2_O_2_ and MDA concentrations | Improved seed germination | + | [49] |
| *Capsicum* *annuum* | Seeds | Primming with SA | Higher germination rate | + | [107] |
| Pomegranate | Leaves | Salinity | Decreased the net photosynthetic rate, chlorophyll content, stomatal conductance, relative water content, and electrical conductivity | - | [114,118,119] |
| Citrus | Intermediate and young leaves | External application of ABA under salinity stress | Reduced ethylene concentration and leaf abscission | + | [165] |
| Soyabean | Seedlings | Exogenous application of JA | Salinity tolerance | + | [169] |
